# Hsa_circ_0074298 promotes pancreatic cancer progression and resistance to gemcitabine by sponging miR-519 to target SMOC

**DOI:** 10.7150/jca.62927

**Published:** 2022-01-01

**Authors:** Chen Hong, Wang Lishan, Xie Peng, Lei Zhengqing, Yang Yang, Hu Fangfang, Yu Zeqian, Cheng Zhangjun, Zhou Jiahua

**Affiliations:** 1 Department of Hepatopancreatobiliary Surgery, Zhongda Hospital, School of Medicine, Southeast University, Nanjing, Jiangsu Province, 210009, China.; 2 Department of Hepatopancreatobiliary Surgery, Zhongda Hospital Southeast University, Nanjing, Jiangsu Province, 210009, China.

**Keywords:** hsa_circ_0074298, miR-519d, SMOC2 gene, pancreatic cancer, chemo-resistance

## Abstract

**Objective:** To investigate the expression of hsa_circ_0074298 (circular RNA) and the molecular mechanism that promotes tumor growth and enhances the chemoresistance of pancreatic cancer.

**Methods:** Real-time reverse transcription-PCR was used to detect hsa_circ_0074298 expression in pancreatic cancer. The intracellular localization of hsa_circ_0074298 was determined by RNA *in situ* hybridization. The CCK8 method, colony formation assay, Transwell assay, and flow cytometry were used to evaluate the effects of hsa_circ_0074298 on the proliferation, migration, invasion, cell cycle, apoptosis of pancreatic cancer cells. Bioinformatics analysis and dual luciferase assays were employed to detect the association of hsa_circ_0074298 and miR-519d and the binding of miR-519d to the target gene SMOC2. A subcutaneous xenograft model was established to observe the effect of hsa_circ_0074298 *in vivo*.

**Results:** The hsa_circ_0074298 was mainly localized in the cytoplasm. Hsa_circ_0074298 was highly expressed in pancreatic cancer tissues and cell lines. The expression of hsa_circ_0074298 was significantly correlated with pancreatic cancer tumor size, lymph node metastasis, and pathological grade. hsa_circ_0074298 could sponge miR-519, and miR-519d bound to SMOC2. Downregulation of hsa_circ_0074298 expression significantly inhibited cell proliferation, migration, invasion, colony forming ability and promoted cell cycle arrest, apoptosis and chemo-resistance of pancreatic cancer *in vitro* and *vivo*. However, the effects could be reversed by a miR-519d inhibitor or SMOC2 overexpression.

**Conclusion:** By sponging miR-519 and targeting SMOC2, hsa_circ_0074298 promotes the growth and metastasis of pancreatic cancer and increases the resistance of pancreatic cancer cells to gemcitabine.

## Introduction

Pancreatic cancer is a highly malignant tumor of the digestive system. Early diagnosis is difficult, and the mortality rate is high. Even in patients with early-stage pancreatic cancer, the median survival time after combination therapy is less than 2 years, with 5-year survival rates ranging from 15 to 20% 1. It will become the second most fatal cancer by 2030 1, 2. Chemotherapy-acquired drug resistance is the main issue facing pancreatic cancer patients without surgical opportunities and undergoing postoperative treatments. Therefore, it is of great clinical significance to conduct in-depth research on the molecular mechanism of action of pancreatic cancer and to explore new treatment methods.

Circular RNAs (circRNAs) are a newly identified class of noncoding RNAs. CircRNAs are widely present in eukaryotic cells and account for more than 90% of total RNA in human somatic cells 3. Most circRNAs are transcribed from exons; only a small portion of circRNAs are transcribed from introns 4. Compared with traditional linear RNAs, circRNAs are noncoding RNAs that do not have a 5' cap or a 3' poly(A) tail and are not cleaved by exonucleases 5. Competitive endogenous RNAs (ceRNAs) are considered the main indispensable mechanism by which circRNAs regulate gene expression. CircRNAs have been proven to be microRNA (miRNA) molecular absorption sponges with miRNA binding sites and thereby to indirectly regulate the expression of corresponding target genes 6, 7. The abnormal expression of circRNAs is related to tumor occurrence, invasion, metastasis, and chemoresistance 8. CircRNAs can be used as tumor markers for clinical diagnosis 9. Bladder cancer studies have found that the circRNA ITCH regulates the expression of p21 and phosphatase and tensin homologue (PTEN) by sponging miR-17/miR-224 10; in pancreatic cancer, the circRNA LDLRAD3 can be used as a biomarker for pancreatic cancer diagnosis 11, and circRNA_100782 can regulate the proliferation of pancreatic cancer cells through IL6-STAT3 signaling 12. CircRNAs have become a new research hotspot in the field of oncology.

miRNAs are also a type of noncoding single-stranded small RNA that are widely present in eukaryotic cells. They regulate gene expression and mediate the occurrence and development of tumors. Moreover, some miRNAs are abnormally expressed in tissues, blood, and body fluids and are closely related to the prognosis of diseases. Therefore, they can be used as biomarkers of a variety of cancers 13, 14. Studies have shown that miRNAs can induce tumor cell drug resistance through different mechanisms. For example, miR-181c is upregulated in pancreatic cancer and contributes to pancreatic cancer chemoresistance through the Hippo pathway, resulting in a poor prognosis 15. Studies have confirmed that in pancreatic cancer, miR-519d can inhibit tumor growth by regulating the Wnt/β-catenin signaling pathway 16, and lncRNA plasmacytoma variant translocation 1 (PVT1) can exert tumor suppression effects through the miR-519d/HIF-1α axis 17. Based on bioinformatics prediction using the starBase database (http://starbase.sysu.edu.cn/), miR-519d can target and bind multiple upstream circRNAs, among which hsa_circ_0074298 shows the strongest binding affinity. Therefore, hsa_circ_0074298 and miR-519d may have regulatory roles in tumor growth and chemoresistance in pancreatic cancer. In this study, we designed experiments to verify this hypothesis.

The miRNA target gene prediction database TargetScanHuman (http://www.targetscan.org/) predicts that the possible target gene of miR-519d is secreted protein acidic and rich in cysteine (SPARC)-related modular calcium-binding protein 2 (SMOC2), which, as a SPARC family member, is highly expressed during wound healing and embryogenesis 18-20. The SMOC2 gene product is a protein that stimulates endothelial cell migration and proliferation and angiogenesis activity 21, 22. Studies have shown that targeting the tumor stem cell marker gene SMOC2 can inhibit the proliferation of endometrial cancer cells and overcome chemoresistance 23. SMOC2 can also promote cancer cell proliferation by regulating cell cycle progression 24, 25.

In this study, we aimed to confirm that hsa_circ_0074298 can promote the proliferation and metastasis of pancreatic cancer cells and to explore the regulatory effect of hsa_circ_0074298 on the miR-519d/SMOC2 axis.

## Materials and methods

### Human pancreatic tissue specimens

From January 2018 to June 2019, cancer and paracancerous tissue specimens from 30 patients with pancreatic cancer undergoing surgical treatment at the Department of Hepatic-Biliary-Pancreatic Center, Zhongda Hospital Southeast University, China, were collected. None of the pancreatic cancer patients underwent chemotherapy, radiotherapy, or targeted therapies before surgery, and informed consent for the collection of clinicopathological data was obtained from all patients. The study was approved by the ethics committee of Zhongda Hospital Southeast University (NO.: 2016ZDSYLL027.0.). Tumor-node-metastasis (TNM) staging was based on the 8th edition of the American Joint Committee on Cancer (AJCC).

### Cells and cell culture

Human pancreatic cancer cell lines (PANC-1 (3101HUMSCSP535), BxPC-3 (3101HUMTCHu012), SW1990 (1101HUM-PUMC000472) and AsPC-1 (1101HUM-PUMC000214) cells), purchased from Shanghai Institute of Biochemistry and Cell Biology, Chinese Academy of Sciences, were cultured in DMEM (HyClone, USA) containing 10% fetal bovine serum (Thermo Fisher, USA) and 100 U/mL penicillin and streptomycin (HyClone, USA). A normal human pancreatic duct epithelial (HPDE) cell line, a gift from Professor Ming-Sound Tsao (Toronto General Hospital, Toronto Western Hospital, Princess Margaret Hospital, University Health Network), was cultured in keratinocyte SFM + EGF + bovine pituitary extract serum-free medium (Invitrogen) containing 1× antibiotic-antimycotic (Gibco). All cells were cultured with 5% CO_2_ at 37°C.

The constructed gemcitabine-resistant human pancreatic cancer cell line PANC-1 in logarithmic growth phase was diluted to a cell suspension of 1×10^6^ cells/mL, and 1 mL of the cell suspension was inoculated in medium with gemcitabine at a final concentration of 10 nM. When the cells could maintain growth and the survival rate was higher than 90%, the concentration of gemcitabine was gradually increased, and ultimately, the cells were cultured in gemcitabine-containing (200 μM) medium. The obtained gemcitabine-resistant strain, PANC-1-GEM, showed stable resistance to a high concentration of gemcitabine and maintained stable resistance when cultured in gemcitabine-free medium.

### Cell transfection and transduction

To construct a stably transduced hsa_circ_0074298 interference cell line, lentiviral vectors containing 3 hsa_circ_0074298 sequence interference plasmids and an empty control plasmid were constructed (Shanghai GenePharma Co., Ltd.). The sequences were as follows: shcirc-1: 5'-CCACTCGGTATTTGGCAATGAATA-AACTTCAAGAGAGTTTATTCATTGCCAAATACCGA-3', shcirc-2: 5'-CACCGG-AACTTTGATCCCATGATCCTTCAAGAGAGGATCATGGGATCAAAGTTCC-3', shcirc-3: 5'-CACCGCTGCTAGAGGAAAGACTAAATTCAAGAGATTTAGTCTT-TCCTCTAGCAGC-3', and sh-NC: 5'-GATCCCCTAAGGTTAAGTCGCCCTC-GCTCGAGCGAGGGCGACTTAACCTTAGGTTTTTG-3'.

Pancreatic cancer cells (AsPc-1, BxPC-3, PANC-1, SW1990) in logarithmic growth phase were prepared as a cell suspension with a cell density of 2×10^5^/mL, and 1 mL of cell suspension was inoculated into each well of a 24-well plate and cultured for 24 h in an incubator. The original medium was replaced with fresh medium containing 6 μg/mL polybrene, and ultracentrifuged viral solutions were added to the cells for transduction and screening based on the virus titers of the hsa_circ_0074298 interference virus (sh-circ0074298) and NC (sh-NC) virus provided by Shanghai GenePharma Co., Ltd. Complete medium containing 800 μg/mL G418 was used for selection. The hsa_circ_0074298 target sequence with the best inhibitory effect was selected for stable transduction, single-cell colony screening, and cell passage. G418 (200 μg/mL) was used for selection maintenance. After 3 passages, stably transduced strains were obtained for later use or cryopreservation. The same method was used to construct gemcitabine-resistant PANC-1 cell lines (PANC-1-GEM) that stably expressed sh-circ0074298 or sh-NC.

Plasmids containing the miR-519d inhibitor antagonist miR (5′-CUCUAGAGGGAAGCGCUUUCUG-3), negative control stable NC mimic (5′-GUUUCACGGAGGGAAAUCUCAC-3′), and SMOC2 (Shanghai GenePharma Co., Ltd.) were constructed and transiently transfected into stable cell lines, i.e., sh-circ0074298-PANC-1, sh-circ0074298-BxPC-3, and sh-circ0074298-PANC-1-GEM, using Lipofectamine 3000 (Invitrogen, USA). Full-length hsa_circ_0074298 was subcloned into the lentivirus vector (ov-circ0074298) by GenePharma (Shanghai, China) following the manufacturer's protocol.

### Real-time reverse transcription-PCR (qRT-PCR)

Human pancreatic cancer tissue, paracancerous tissue, or mouse cancer tissue was weighed (50-100 mg), ground thoroughly, and transferred to an RNase-free centrifuge tube. All pancreatic cancer cells and normal HPDE cells were cultured to 80-90% confluence and rinsed twice with PBS prechilled to 4°C. Total RNA was extracted according to the instructions provided with the TRIzol RNA kit (Invitrogen, USA).

Extraction of miRNA from cancer cells and tissue specimens was carried out following the instructions provided with the EasyPure® miRNA Kit (TransGen Biotech Co., Ltd., Beijing, China).

Total RNA was reverse transcribed to cDNA following the instructions provided with the RevertAid TM First Strand cDNA Synthesis Kit (Thermo Scientific TM, USA). The cDNA was diluted to 50 ng/μL. The primers were designed and synthesized by Shanghai GenePharma Co., Ltd. The primer sequences are provided in Table 1. The final concentration of the primers was 0.5 μM, the total volume of the reaction system was 25 μL, and the reaction parameters were as follows: predenaturation at 95°C for 180 sec; 45 cycles of 95°C for 5 sec, 95°C for 10 sec, and 60°C for 45 sec; and annealing at 60°C for 45 sec.

The primers for miR-519d qRT-PCR were designed and synthesized by Shanghai GenePharma Co., Ltd.: miR-519d RT primer: GTCGTATCCAGTGCGTGTCG-TGGAGTCGGCAATTGCACTGGATACGACCACTCT; miR-519d forward primer: 5'- CAAAGTGCCTCCCTTT-3', and reverse primer: 5'- CAGTGCGTGTCGTGGAGT-3'; and U6 forward primer: 5'-CTCGCTTCGGCAGCACATA-3', and reverse primer: 5'-AACGATTCACGAATTT-GCTG-3'. Twelve microliters of miRNA reverse transcription primers and U6 reverse transcription primers were mixed well separately and added to 96 μL of RNase-free H_2_O to obtain a 1 μM reverse transcription primer working solution. In accordance with instructions provided with the One Step PrimeScript® miRNA cDNA Synthesis Kit (Bao Biological engineering (Dalian) Co., Ltd.), a reverse transcription reaction system was established with a total volume of 20 μL and reaction setting of 37°C for 60 min, 50°C for 60 min, and 85°C for 5 sec. After the reaction, the product was immediately placed on ice. Following the instructions provided with the SYBR® Premix Ex Taq II kit (Takara, Japan), the total volume of the reaction system was 20 μL, and the reaction parameters were as follows: predenaturation at 95°C for 3 min; 40 cycles of 95°C for 12 sec and 60°C for 30 sec; and annealing at 62°C for 40 sec.

### RNA *in situ* hybridization

An hsa_circ_0074298 RNA *in situ* hybridization probe was synthesized by Shanghai GenePharma Co., Ltd.; the sequence was as follows: 5'-GCCTCAACCACGGAGTTCCTTTTGCTCGG-3'. Paraffin sections of human pancreatic cancer tissue and paracancerous tissue were processed in accordance with the instructions provided by the RNA FISH probe kit F04302 (Shanghai GenePharma Co., Ltd.). The tissue sections were observed under a fluorescence microscope, and images were collected and recorded.

### CCK-8 assay

Pancreatic cancer cells were inoculated into a 96-well plate at 2000 cells/well, with at least 5 replicate wells in each group. After culturing for 24 h, 48 h, or 72 h, cell proliferation was determined following the recommended procedure of the CCK-8 kit (Dojindo, Japan): 10 μL of CCK-8 was added to each well, and the cells were cultured for another 1 h; a blank well was used to zero the instrument, and the OD_450_ value was measured. The growth curve for pancreatic cancer cells was graphed with the detection time as the horizontal axis and the absorbance value as the vertical axis (OD_450_) to compare the growth rates of cancer cells in each group.

### Flow cytometry

Transfected pancreatic cancer cells were seeded at 1.5×10^6^ cells per well in a 6-well plate; 3 replicate wells were set up concurrently. The cells were harvested after culturing to 70-80% confluence, washed with PBS, fixed in 70% ethanol at 4°C overnight, rinsed with PBS, and treated in accordance with the instructions provided with the Cycle TESTTM PLUS DNA Reagent Kit (BD Biosciences, USA). The cell cycle distribution for each group of cells was detected using flow cytometry. For the apoptosis assay, all cells (including dead cells) were collected, washed twice with PBS, resuspended in 1× binding buffer to a cell density of 10^6^ cells/mL, and processed in accordance with the instructions provided with the PE Annexin V Apoptosis Detection Kit I (BD Biosciences, USA) for apoptosis analysis using flow cytometry.

### Transwell assay

First, complete medium was added to a 24-well plate (500 μL/well), a Transwell chamber was placed on each well, and the plate was incubated in a cell culture incubator for 30 min. The cells were digested, resuspended, and counted. The cell suspension was adjusted to 5×10^4^ cells/mL with DMEM only, and 100 μL of the cell suspension was added to each chamber; 3 replicate wells were set up for each group. The 24-well plate was placed in an incubator, and the chambers were removed after 24 h to be stained with crystal violet to observe cell migration. For the invasion assay, Matrigel and ice-cold DMEM were mixed at a ratio of 1:8 into a Matrigel DMEM mixture in advance, which was placed on ice for later use.

### Colony formation assay

Pancreatic cancer cells in logarithmic growth phase were digested with trypsin to prepare a cell suspension containing at least 95% single cells. Fifty pancreatic cancer cells were inoculated into each well of a 12-well plate, and the cells were evenly dispersed in the medium. When white cell clumps appeared in the culture plate, the cell culture was stopped, and the plate was washed twice with PBS; the cells were dried, fixed with methanol for 15 min, dried, stained with 1% crystal violet for 15-30 min, rinsed to remove the staining solution, and dried again. The number of single colonies with more than 50 cells was counted under a microscope to compare differences in the colony forming ability of each group.

### Dual luciferase assay

The hsa_circ_0074298 sequence containing miR-519d, miR-582-3p, miR-510, miR-497, miR-503, miR-515-3p, miR-532-3p, miR- 572, miR-578, miR-548p, miR-615-5p, and miR-769-3p binding sites and a mutated sequence (WT/MUT) were used to construct dual luciferase reporter gene vectors (GenePharma Co., Ltd., Suzhou, China), which were cotransfected into 293 cells with each of the above miRNA mimics using Lipofectamine 3000 transfection reagent. Forty-eight hours after transfection, luciferase activity was detected. Additionally, the SMOC2 sequence containing the miR-519d binding site and a mutated sequence (WT/MUT) were used to construct dual luciferase reporter gene vectors, which were cotransfected into 293 cells with miR-519d mimics. Forty-eight hours after transfection, luciferase activity was detected (GenePharma Co., Ltd., Suzhou, China).

### Western blot analysis

Total protein in human pancreatic cancer tissue and pancreatic cancer cells was extracted using a cell lysis mixture containing cell lysis reagent (Beyotime Biotech, China) and protease inhibitors, and the protein concentration was measured using a BCA kit (Beyotime Biotech). Equal amounts of protein (20 µg) were separated on a 10% SDS-PAGE gel and transferred to a polyvinylidene fluoride (PVDF) membrane. After blocking, the membrane was incubated with primary antibodies: rabbit anti-human SMOC2 polyclonal antibody (1:10000, Abcam, USA), mouse anti-human glyceraldehyde-3-phosphate dehydrogenase (GAPDH) monoclonal antibody (1:5000, Abcam, USA), and mouse anti-human MDR1 monoclonal antibody (1:1000, Santa Cruz, USA). Next, the membrane was incubated with horseradish peroxidase (HRP)-conjugated goat anti-mouse IgG secondary antibody (1:5000, Abcam, USA) or HRP-conjugated goat anti-rabbit IgG secondary antibody (1:10000, Abcam, USA) and was exposed after developer treatment. For the quantitative analysis, ImageJ 1.51j8 was used to analyze the gray value of all bands.

### Subcutaneous xenograft model

Seventy-six nude mice (BALB/c/nu) were purchased from Shanghai Model Organisms Center, Inc., China; the sex ratio was approximately 1:1, and the mice were approximately 3-5 weeks old, with a body weight of 18-21 g. The mice were bred in a special pathogen-free (SPF) environment with constant temperature (25-27°C) and constant humidity (relative humidity of 45-50%). All animal experiments and operations were approved by the Animal Ethics Committee of Southeast University School of Medicine.

Human pancreatic cancer cell suspension (0.3 mL; cell density of 10^7^/mL) was injected into the sterilized skin on the back of the nude mice. Obvious skin bumps were observed. 5 days after transplantation, tumors on the backs of nude mice had grown to a size larger than 5 mm, suggesting that a pancreatic cancer subcutaneous xenograft model in nude mice was successfully established. The volume of the tumor was measured with a Vernier caliper every 5 days and calculated using the following formula: 0.5 × length × width^2^; the growth curves for tumors were graphed accordingly. On Day 30, the mice were sacrificed by cervical dislocation, the tumors were removed and weighed, and the tumors were placed in RNase-free cryopreservation tubes and stored in liquid nitrogen for later use.

### Statistical analysis

The Student's t test, chi-squared test, one-way analysis of variance, and Kendall correlation analysis were used. The data are expressed as the mean ± standard deviation (mean ± SD). R-3.6.3 was used for data analysis, and GraphPad Prism 7 was used for data analysis and graphing. P<0.05 was considered to indicate a significant difference.

## Results

### Expression of hsa_circ_0074298 was upregulated in pancreatic cancer tissue

**Figure 1:**
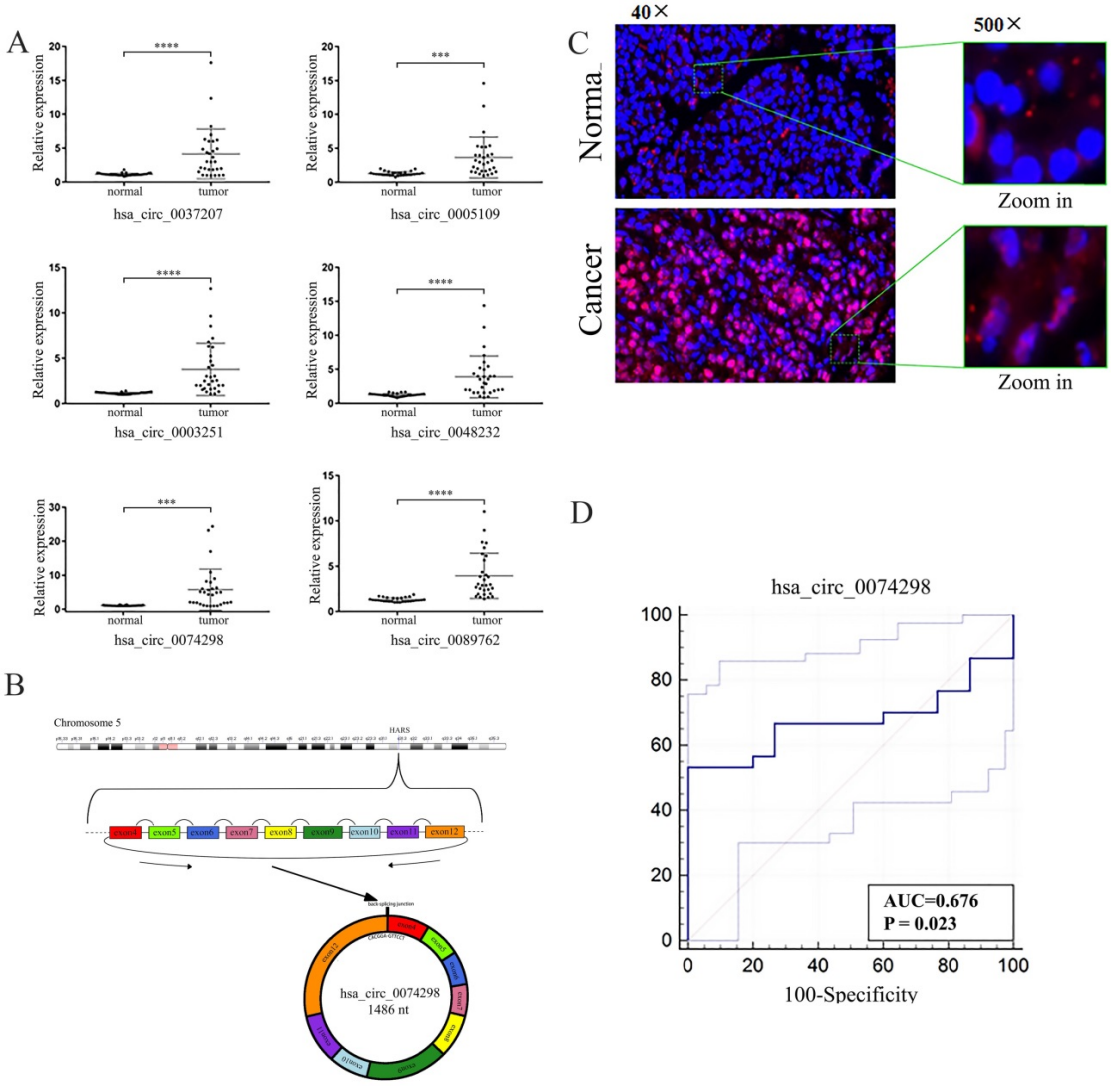
Expression and localization of hsa_circ_0074298 RNA in pancreatic cancer tissue and paracancerous tissue. (A) Expression of hsa_circ_0037207, hsa_circ_0005109, hsa_circ_0003251, hsa_circ_0048232, hsa_circ_0074298, and hsa_circ_0089762 in pancreatic cancer tissue and paracancerous tissue (*, vs normal). (B) Genomic locus of hsa_circ_0074298; (C) Expression and intracellular sublocalization of hsa_circ_0074298 verified by RNA *in situ* hybridization. (D) ROC curve for hsa_circ_0074298. ***P<0.001, ****P<0.0001.

The expression of 6 circRNAs, i.e., hsa_circ_0037207, hsa_circ_0005109, hsa_circ_0003251, hsa_circ_0048232, hsa_circ_0074298, and hsa_circ_0089762, was significantly higher in human pancreatic cancer tissue than in paracancerous tissue (FC>2.5, P<0.001) (Table 2, Figure 1A). The difference in expression of hsa_circ_0074298 was the most significant (FC=5.23, P<0.01). Hsa_circ_0074298 was derived from exons 4-12 of the HARS gene (chr5: 140053489- 140058712) (Figure 1B). The results of the RNase R treatment assay showed that, compared with linear β-actin, hsa_circ_0074298 was recalcitrant to RNase R-mediated degradation (Figure S1). The expression of hsa_circ_0074298 in pancreatic cancer tissue was also significantly higher than that in paracancerous tissue, and based on RNA *in situ* hybridization, it was mostly localized in the cytoplasm (Figure 1C).

The expression of hsa_circ_0074298 was significantly increased in pancreatic cancer tissue and was correlated with tumor size (Table 3). The Kendall correlation analysis of hsa_circ_0074298 expression in pancreatic cancer tissue and the clinicopathological data from the patients indicated that hsa_circ_0074298 expression was significantly correlated with tumor diameter (P=0.024) and pathological grade (P=0.012). Further multivariate analysis using a multivariate generalized linear regression model showed that hsa_circ_0074298 expression was related to tumor diameter, lymphatic metastasis, and pathological grade (Table 5). The diagnostic efficacy of the independent application of hsa_circ_0074298 as a diagnostic marker showed an area under the receiver operating characteristic (ROC) curve of 0.676 (P=0.023), a 95% confidence interval of 0.542-0.791, a sensitivity of 0.667 (0.472- 0.827), and a specificity of 0.733 (0.541-0.877) (Figure 1D), indicating that hsa_circ_0074298 could be used as a marker for the diagnosis of pancreatic cancer.

### Expression of hsa_circ_0074298 was upregulated in human pancreatic cell lines

**Figure 2:**
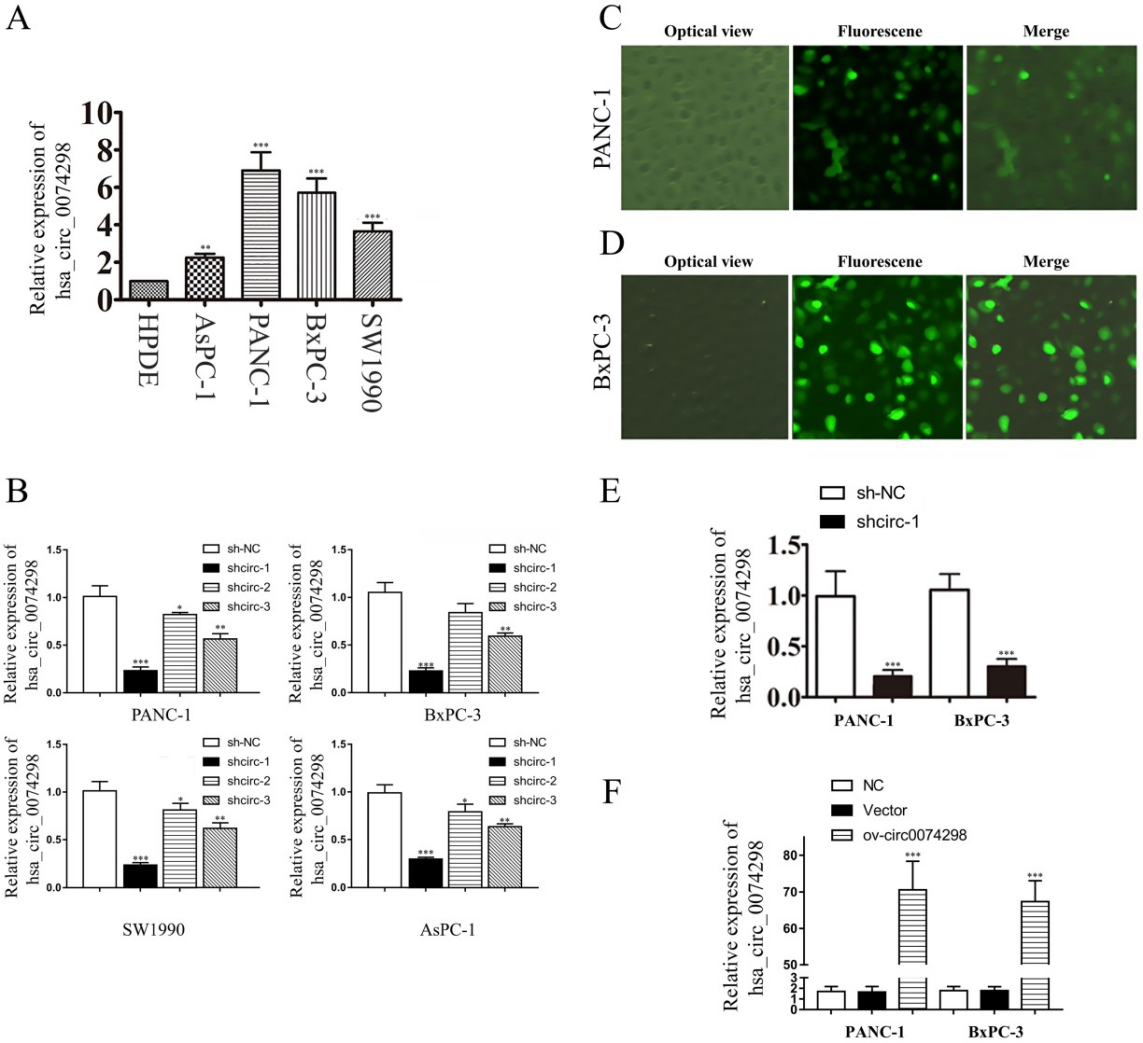
hsa_circ_0074298 expression in human pancreatic cell lines and construction of human pancreatic cancer cells stably transduced with sh-circ0074298 or ov-circ0074298. (A) Expression of hsa_circ_0074298 in human pancreatic cancer cells and normal pancreatic cells (*, vs HPDE). (B) Expression of hsa_circ_0074298 in pancreatic cancer cells after transduction of shcirc-1, shcirc-2, and shcirc-3 (*, vs sh-NC). (C, D) PANC-1 and BxPC-3 cell status after transient lentivirus transduction. (E) Expression of hsa_circ_0074298 expression in shcirc-1 stably transfected PANC-1 and BxPC-3 cells (*, vs sh-NC). (F) Expression of hsa_circ_0074298 in ov-circ0074298 cancer cells (*, vs Vector). **P<0.01, ***P<0.001.

qRT-PCR was used to detect the expression of hsa_circ_0074298 in PANC-1, SW1990, AsPC-1, BxPC-3, and HPDE cells. The results showed that hsa_circ_0074298 expression was significantly higher in human pancreatic cancer cells than in HPDE cells (Figure 2A). PANC-1 and BxPC-3 cells, with the highest expression level of hsa_circ_0074298, were selected for subsequent experiments.

### Validation of the transduction efficiency of hsa_circ_0074298 interference vectors and construction of stable transduced cell lines

Plasmids containing the sh-circ0074298 or sh-NC sequence were transduced into PANC-1, BxPC-3, SW1990, and AsPC-1 cells by lentiviral infection, and 3 sh-circ sequences (shcirc-1, shcirc-2, and shcirc-3) were designed. The results indicated that shcirc-1 showed the greatest downregulation efficacy in all 4 pancreatic cancer cell lines (Figure 2B). The interference results for sh-circ0074298 transient transduction detected by qRT-PCR showed an optimal interference efficiency of shcirc-1 in PANC-1 (Figure 2C) and BxPC-3 cells (Figure 2D), demonstrating the most significant downregulation effect on the expression of hsa_circ_0074298. After screening, PANC-1 and BxPC-3 cells stably transduced with shcirc-1 (Figure 2E) or ov-circ0074298 (Figure 2F) were obtained. The Western blot results showed that downregulation of hsa_circ_0074298 exerted no significant impact on the expression of HARS (Figure S2).

### hsa_circ_0074298 promoted the proliferation and metastasis of human pancreatic cancer cells

**Figure 3:**
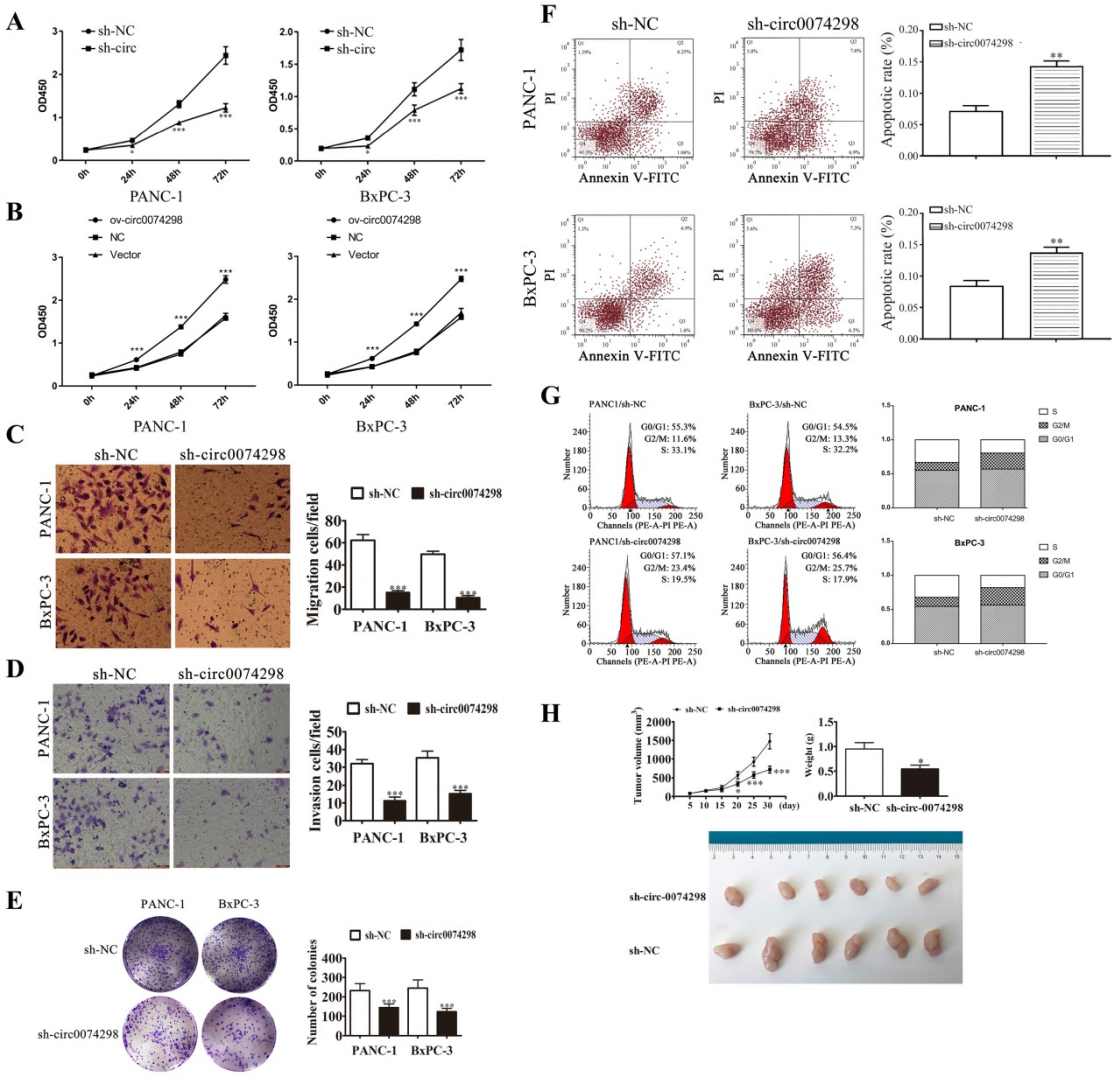
hsa_circ_0074298 affected the biological behavior of human pancreatic cancer cells. (A) Effects of hsa_circ_0074298 downregulation on the proliferation of pancreatic cancer cells (*, vs sh-NC). (B) Effects of hsa_circ_0074298 upregulation on the proliferation of pancreatic cancer cells (*, vs Vector). (C) Transwell assay to detect the migration abilities of PANC-1 and BxPC-3 cells (*, vs sh-NC). (D) Transwell assay to detect the invasion abilities of PANC-1 and BxPC-3 cells (*, vs sh-NC). (E) Colony formation assay to detect PANC-1 and BxPC-3 cell colony formation (*, vs sh-NC). (F) Changes in apoptosis of PANC-1 and BxPC-3 cells after hsa_circ_0074298 knockdown (*, vs sh-NC). (G) Changes in the cell cycle of PANC-1 and BxPC-3 cells after hsa_circ_0074298 knockdown. (H) The subcutaneous xenograft volume (Days 0, 5, 10, 15, 20, 15, and 30 after transplantation) and weight (Day 30 after transplantation) of excised tumors (N=6/group) (*, vs sh-NC). *P<0.05, **P<0.01, *** P<0.001.

A CCK8 kit was used to detect the proliferation of pancreatic cancer cells (PANC-1 and BxPC-3) stably transduced with sh-circ0074298 or sh-NC at 24 h, 48 h, and 72 h, respectively. The results showed that after interfering with hsa_circ_0074298 expression, compared with the control group, the proliferation of PANC-1 and BxPC-3 cells decreased significantly at 24 h, 48 h, and 72 h (P<0.05) (Figure 3A). Overexpression of hsa_circ_0074298 significantly promoted the proliferation of pancreatic cancer cells at 24 h, 48 h, and 72 h (P<0.001) (Figure 3B). Transwell assays to detect the migration and invasion ability of cancer cells indicated that after downregulating hsa_circ_0074298 expression, the migration ability (Figure 3C) and invasion ability (Figure 3D) of PANC-1 and BxPC-3 cells decreased significantly after 24 h of incubation. After downregulating the expression of hsa_circ_0074298, the ability of PANC-1 and BxPC-3 cells to form colonies also decreased significantly (Figure 3E).

In PANC-1 and BxPC-3 cells stably transduced with sh-NC or sh-circ0074298, flow cytometry detection of the apoptosis ratio indicated that the proportion of apoptotic cells was significantly higher in PANC-1 and BxPC-3 cells stably transduced with sh-circ than sh-NC (P<0.01) (Figure 3F), indicating that hsa_circ_0074298 had a significant effect on promoting pancreatic tumor cell apoptosis. Further detection of cell cycle changes in pancreatic tumor cells after interference with hsa_circ_0074298 showed that in PANC-1 and BxPC-3 cells stably transduced with sh-circ, G2/M phase cells increased while S phase cells decreased (Figure 3G), indicating that the downregulation of hsa_circ_0074298 expression could induce G2/M arrest in pancreatic cancer cells.

Five days after nude mice were transplanted with PANC-1 cells that were stably transduced with sh-circ0074298 or sh-NC, irregular masses with a diameter of 4-6 mm were observed on the back skin, confirming the successful establishment of a xenograft tumor model. Tumor parameters were recorded at fixed time points, and the tumors were removed on Day 30 after cell transplantation. The results showed that the tumor volume and weight were significantly lower in the sh-circ0074298 group than in the sh-NC group (P<0.01). As observed by the naked eye, the surface of the removed tumors was irregular, the texture was hard, and the color of the cut surface of tumors was grayish white (Figure 3H).

### hsa_circ_0074298 regulated the expression of SMOC2 and affected the biological behavior of pancreatic cancer by sponging miR-519d

**Figure 4:**
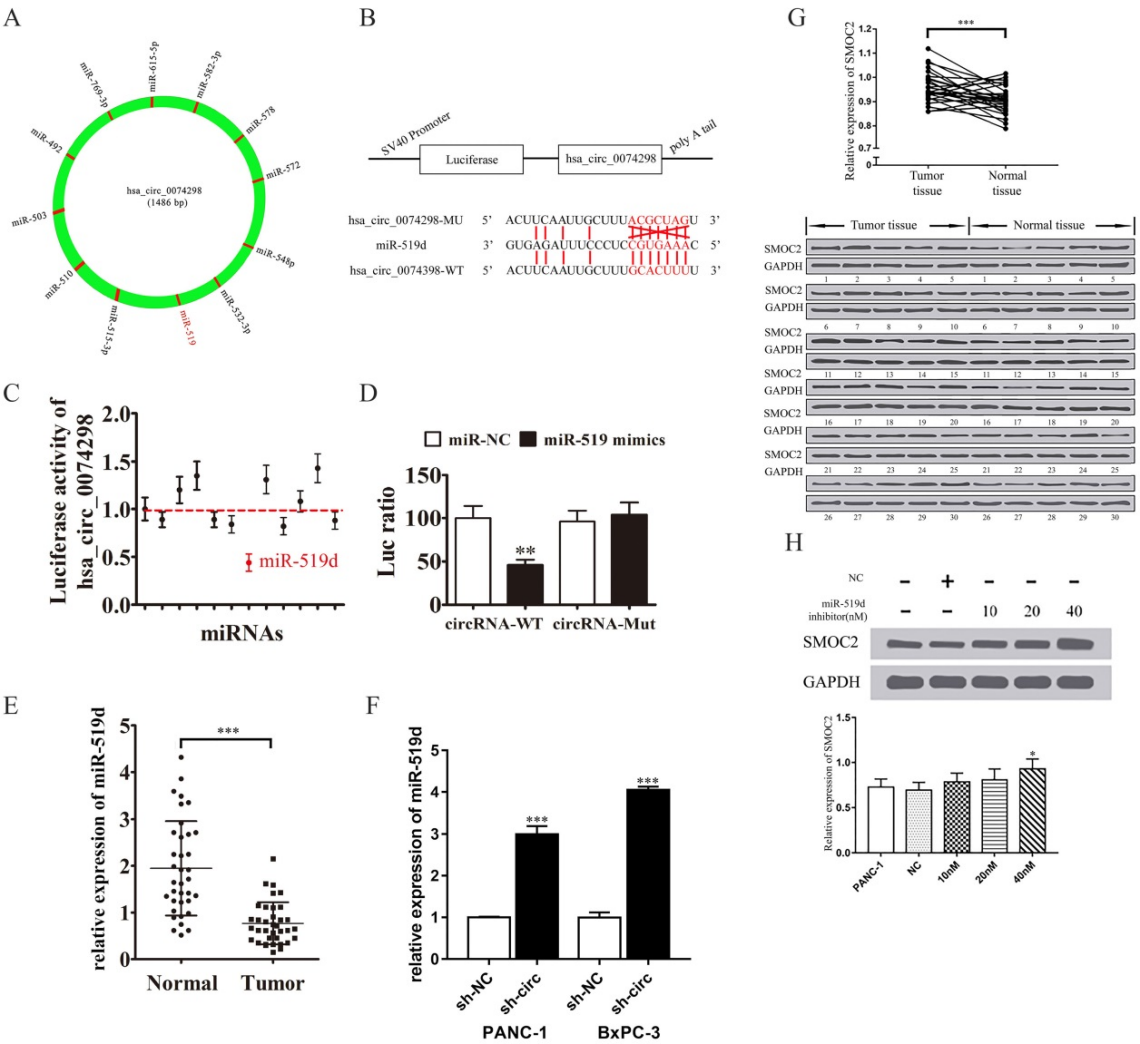
hsa_circ_0074298 sponged miR-519/SMOC2. (A) hsa_circ_0074298 sponged miRNA. (B) The sequences of hsa_circ_0074298 and miR-519d used in the dual luciferase reporter assay. (C) The fluorescence activity of the predicted miRNA binding sites of hsa_circ_0074298 verified by the dual luciferase assay. (D) The fluorescence activity of the miR-519d binding site of hsa_circ_0074298 verified by the dual luciferase assay (*, vs miR-NC). (E) qRT-PCR detection of miR-519d expression in pancreatic cancer tissue and normal pancreatic tissue (*, vs Normal). (F) qRT-PCR detection of miR-519d expression in PANC-1 and BxPC-3 cells stably transduced with sh-NC or sh-circ0074298 (*, vs sh-NC). (G) Western blot detection of SMOC2 expression in pancreatic cancer and normal pancreatic tissues (*, vs Normal tissue). (H) Effect of transfection of different doses of miR-519d inhibitor on SMOC2 protein expression (*, vs sh-NC). *P<0.05, **P<0.01, ***P<0.001.

An online database predicted that hsa_circ_0074298 might sponge miRNAs, including miR-492, miR-503, miR-510, miR-515-3p, miR-519d, miR-532- 3p, miR-548p, miR-572, miR-578, miR-582-3p, miR-615-5p, and miR-769-3p (Figure 4A, B, Table S1). Further dual luciferase reporter gene detection results showed that the 3'UTR sequence (WT) of hsa_circ_0074298 bound with all 12 miRNA mimics, generating fluorescence activity (Figure 4C), the fluorescence activation degree of which was lowest between the 3'UTR sequence of hsa_circ_0074298 and miR-519d, which indicated that they had the strongest binding capacity (P<0.01). Moreover, a comparison of the fluorescence activity after the WT or MUT 3'UTR sequence of hsa_circ_0074298 bound to miR-519d mimics (Figure 4D) indicated that the WT 3'UTR sequence of hsa_circ_0074298 and miR-519d produced a lower degree of fluorescence activation (P<0.01).

qRT-PCR detection of miR-519d expression in pancreatic cancer tissue and in PANC-1 and BxPC-3 cells stably transduced with sh-NC or sh-circ0074298 showed that miR-519d expression was significantly lower in pancreatic cancer tissue than in normal pancreatic tissue (P<0.001) and that expression of miR-519d was significantly higher in PANC-1 (P<0.001) and BxPC-3 (P<0.001) cells stably transduced with sh-circ0074298 than that in sh-NC control cells (Figure 4E, F).

Western blotting was used to detect the protein expression of SMOC2 in 30 pairs of pancreatic cancer tissue and corresponding paracancerous tissue samples. The results showed that SMOC2 was highly expressed in pancreatic cancer tissue (P<0.001) (Figure 4G) and was negatively correlated with miR-519d expression.

Subsequently, 10 nM, 20 nM, and 40 nM miR-519d inhibitor or a negative control were transfected into PANC-1 cells. After the transfected cells were cultured for 2 days, the expression of SMOC2 protein was detected by Western blotting. The results showed that with the increase in miR-519d inhibitor concentration, the expression of SMOC2 protein rose (P<0.05) (Figure 4H), suggesting that inhibition of miR-519d promoted the expression of SMOC2.

**Figure 5:**
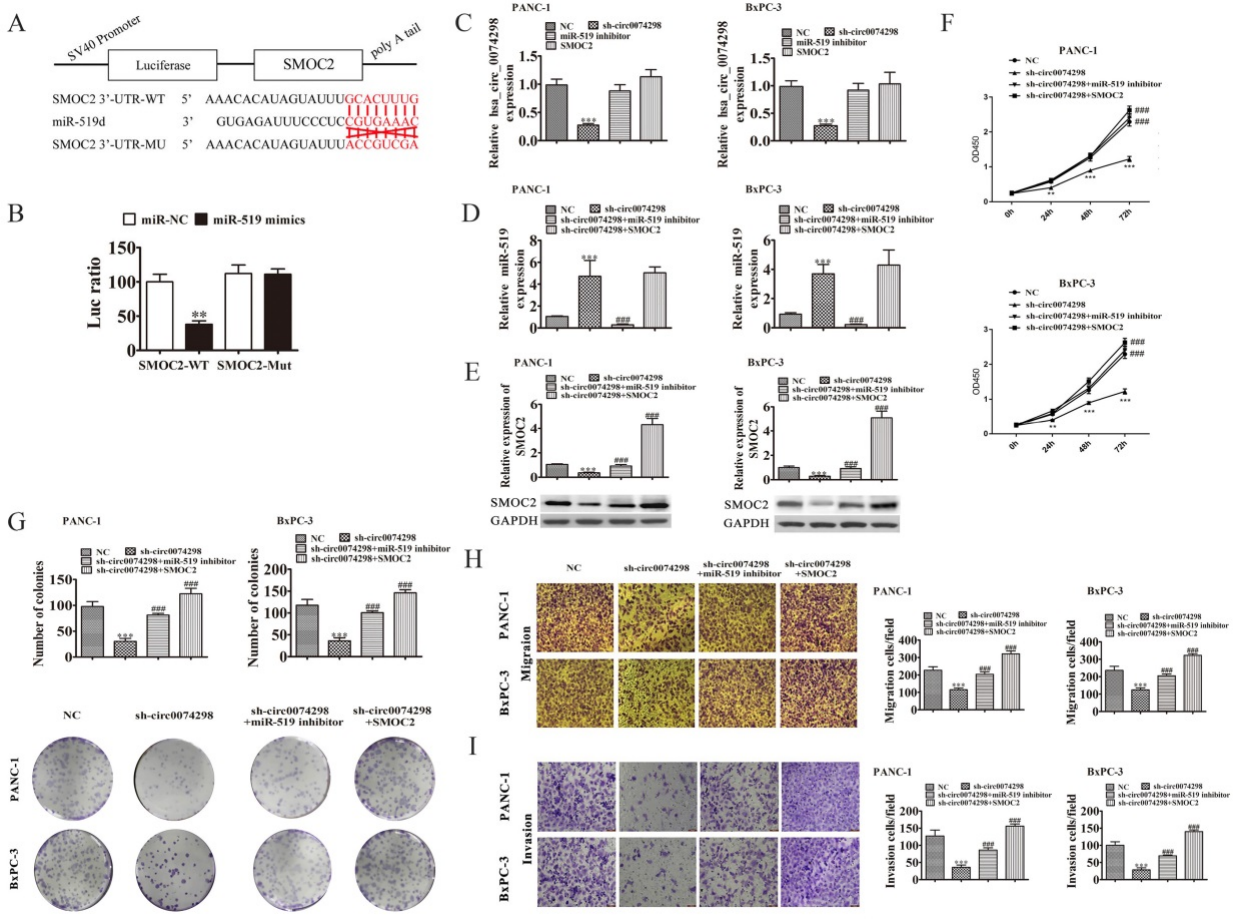
hsa_circ_0074298 regulated the expression of SMOC2 by sponging miR-519d. (A) The sequences of miR-519d and SMOC2 used in the dual luciferase reporter assay; (B) Dual luciferase reporter detection of the binding capability of miR-519d to the SMOC2 3'UTR (*, vs miR-NC); (C)hsa_circ_0074298 expression (*, vs NC, #, sh-circ0074298); (D) miR-519d expression (*, vs NC, #, sh-circ0074298); (E) SMOC2 protein expression(*, vs NC, #, sh-circ0074298); (F) Cell proliferation changes(*, vs NC, #, sh-circ0074298); (G) Cell colony forming ability(*, vs NC, #, sh-circ0074298); (H, I) Cell migration and invasion ability(*, vs NC, #, sh-circ0074298). **P<0.01, ***P<0.001, ###P<0.001.

The possible target gene of miR-519d is SMOC2. A dual luciferase reporter gene assay confirmed that the 3'UTR sequence (WT) of SMOC2 and miR-519d generated the lowest degree of fluorescence activation, suggesting a significant binding capacity between miR-519d and the SMOC2 3'UTR sequence (P<0.01) (Figure 5A, B).

A miR-519d inhibitor or SMOC2 overexpression plasmid was transfected into PANC-1 and BxPC-3 cells stably transduced with sh-circ0074298 or sh-NC, and the expression levels of hsa_circ_0074298 and miR-519d were detected by qRT-PCR. The results showed that the expression of hsa_circ_0074298 was significantly higher in the sh-circ0074298+miR-519d inhibitor group and sh-circ0074298+SMOC2 overexpression group than in the sh-circ0074298 group (P<0.001), but it did not differ from that in the sh-NC control group (Figure 5C). The expression of miR-519d was significantly lower in the sh-circ0074298+miR-519d inhibitor group than in the sh-circ0074298 group (P<0.001), significantly higher in the sh-circ0074298+SMOC2 overexpression group than in the sh-circ0074298+miR-519d inhibitor and sh-NC group (P<0.001), and no different from that in the sh-circ0074298 group (Figure 5D), which indicated that SMOC2 overexpression had no effect on the expression of miR-519d. The expression of SMOC2 was significantly higher in the sh-circ0074298+miR-519d inhibitor group than in the sh-circ0074298 group, and the expression of SMOC2 was significantly lower in the sh-circ0074298 group than in the sh-NC control group (P<0.001) (Figure 5E), indicating that the expression of SMOC2 was regulated by miR-519d.

After miR-519d inhibitor or SMOC2 overexpression plasmids were transfected into PANC-1 and BxPC-3 cells stably transduced with sh-circ0074298 or sh-NC, the CCK8 method was used to detect the proliferation ability of cancer cells. The results showed that in pancreatic tumor cells stably transduced with sh-circ0074298, transfection of miR-519d inhibitor or SMOC2 overexpression plasmids reversed the decrease in cell proliferation caused by the downregulation of hsa_circ_0074298 (P<0.001) (Figure 5F). Colony formation results showed that the miR-519d inhibitor or SMOC2 overexpression reversed the decrease in colony forming ability caused by the downregulation of hsa_circ_0074298 (P<0.001) (Figure 5G). The Transwell chamber assay results indicated that transfection of the miR-519d inhibitor or SMOC2 overexpression plasmids reversed the decrease in cell migration and invasion caused by the downregulation of hsa_circ_0074298 (P<0.001) (Figure 5H, I).

**Figure 6:**
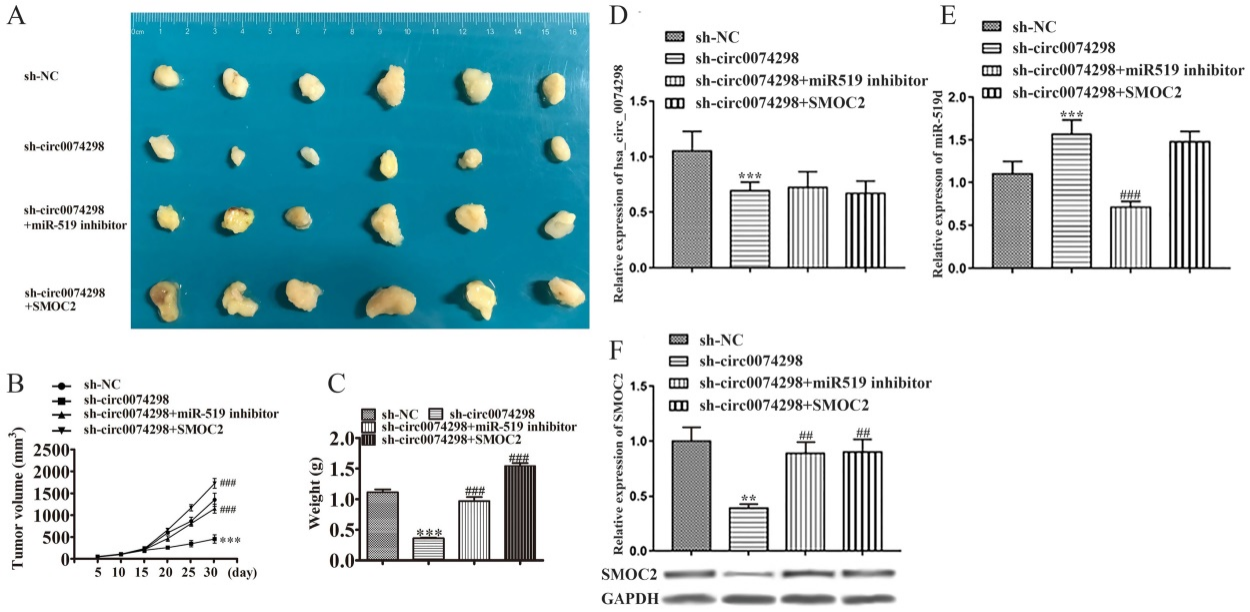
Effect of hsa_circ_0074298 on pancreatic cancer xenografts through the miR-519d/SMOC2 axis *in vivo* (N=6/ group). (A) Image of the tumor. (B) Growth curve. (C) Weight of excised tumors. (D) Expression of hsa_circ_0074298 in tumor tissue. (E) miR-519d expression in tumor tissue. (F) SMOC2 expression in tumor tissue. *, vs sh-NC. #, vs sh-circ0074298. **P<0.01, ***P<0.001, ##P<0.001, ###P<0.001.

After PANC-1 cells stably transduced with sh-circ0074298 or sh-NC empty vectors were transfected with miR-519d inhibitor or SMOC2 overexpression plasmids, a subcutaneous xenograft model using these cells was successfully established. The mice were divided into 4 groups, with 6 mice in each group. Tumor parameters were recorded at fixed time points, and tumor growth curves were graphed. Tumors were removed on Day 30 after transplantation. As observed by the naked eye, the surface of the tumors was irregular, the texture was hard, and the color of the cut surface of tumors was grayish white (Figure 6A). A significantly lower tumor volume and weight were found in the sh-circ0074298 group compared with the sh-NC group (P<0.001); the tumor volume and weight increased significantly in the sh-circ0074298+miR-519d inhibitor and the sh-circ0074298+SMOC2 overexpression groups compared with that in the sh-circ0074298 group (P<0.001) (Figure 6B, C), providing *in vivo* confirmation of the *in vitro* cell experiment results. Transfection of the miR-519d inhibitor or SMOC2 overexpression plasmids could reverse the decrease in cell proliferation ability and cell colony forming ability caused by the downregulation of hsa_circ_0074298.

The expression of hsa_circ_0074298 and miR-519d in tumor tissues was further detected using qRT-PCR. Compared with that in the sh-NC group, hsa_circ_0074298 expression in the sh-circ0074298 group was significantly downregulated (P<0.001), and expression of miR-519d was significantly upregulated (P<0.001) (Figure 6D). Compared with that in the sh-circ0074298 group, hsa_circ_0074298 expression in the sh-circ0074298+miR-519d inhibitor group was not significantly different, while the expression of miR-519d was significantly downregulated (P<0.001) (Figure 6E). Compared with that in the sh-circ0074298 group, the expression of hsa_circ_0074298 and miR-519d in tumor tissues in the sh-circ0074298+SMOC2 group was not significantly different (Figure 6D, E). Western blot analysis revealed SMOC2 protein expression in tumor tissue. Compared with that in the sh-NC control group, SMOC2 protein expression in the sh-circ0074298 group was significantly decreased (P<0.01). Compared with that in the sh-circ0074298 group, SMOC2 protein expression in the sh-circ0074298+miR-519d inhibitor group and sh-circ0074298+SMOC2 overexpression group was significantly upregulated (P<0.01) (Figure 6F).

### Knockdown of hsa_circ_0074298 increased the sensitivity of PANC-1-GEM cells to gemcitabine

**Figure 7:**
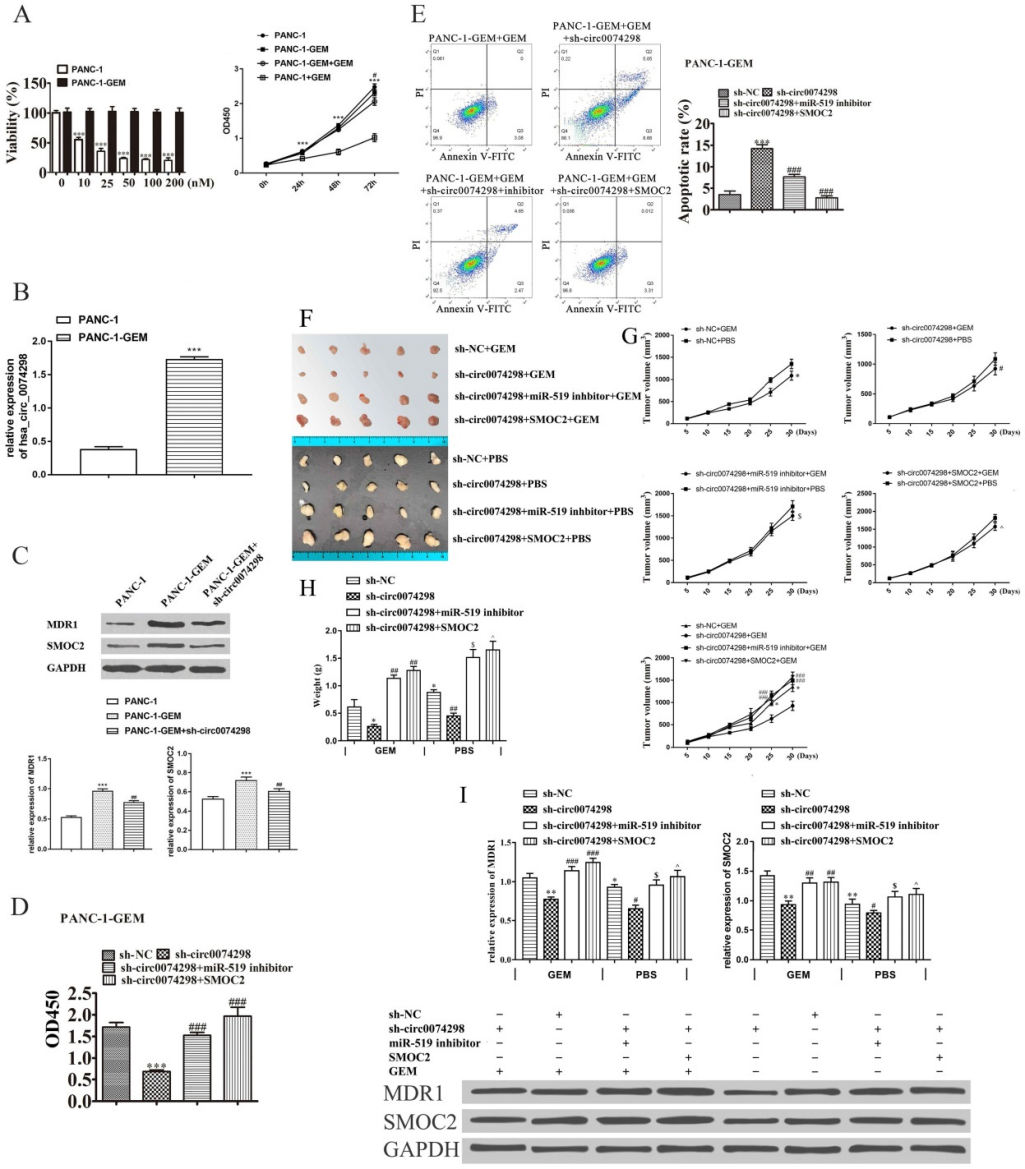
Effect of hsa_circ_0074298 on the chemosensitivity of gemcitabine-resistant PANC-1 cells *in vitro* and *in vivo*. (A) The effect of different concentrations of gemcitabine (GEM) on the proliferation of PANC-1 and PANC-1-GEM cells (*, vs PANC-1; #, vs PANC-1-GEM). (B) hsa_circ_0074298 expression in PANC-1 and PANC-1-GEM cells (*, vs PANC-1). (C) MDR1 and SMOC2 expression in PANC-1 and PANC-1-GEM cells (*, vs PANC-1; #, vs PANC-1-GEM). (D) Changes in cell proliferation after transfection of miR-519d inhibitor or SMOC2 overexpression plasmids into sh-circ0074298 stably transduced PANC-1-GEM cells (#, vs sh-circ0074298). (E) Apoptosis of PANC-1-GEM cells after transfection of miR-519d inhibitor or SMOC2 overexpression plasmids in sh-circ0074298 stably transduced PANC-1-GEM cells (#, vs sh-circ0074298). (F) Representative images of excised tumors from mice subcutaneously inoculated with PANC-1-GEM cells transfected with sh-NC, sh-circ0074298, miR-519 inhibitor, or SMOC2 overexpression plasmids with GEM or PBS intraperitoneal injection (N=5/group). (G) Growth curves of the excised tumors (*, vs sh-NC+ GEM; #, vs sh-circ0074298+ GEM; $, vs sh-circ0074298+miR-519 inhibitor+ GEM; ^, vs sh-circ0074298+ SMOC2+ GEM). (H) Weight of the excised tumors (*, vs sh-NC+ GEM; #, vs sh-circ0074298+ GEM; $, vs sh-circ0074298+miR-519 inhibitor+ GEM; ^, vs sh-circ0074298+ SMOC2+ GEM). (I) Expression of MDR1 and SMOC2 in the excised tumor tissues. (*, vs sh-NC+ GEM; #, vs sh-circ0074298+ GEM; $, vs sh-circ0074298+miR-519 inhibitor+ GEM; ^, vs sh-circ0074298+ SMOC2+ GEM). *P<0.05, **P<0.01, ***P<0.001, #P<0.05, ##P<0.01, ###P<0.001, $P<0.05, ^P<0.05.

Parental PANC-1 cells were cultured using the concentration escalation method (medium containing 0, 10, 25, 50, 100, and 200 nM gemcitabine), ultimately resulting in a PANC-1-GEM-resistant strain. The results showed that compared with the parental strain, the PANC-1-GEM strain showed obvious resistance to gemcitabine and that the proliferation of the parent PANC-1 cells was significantly inhibited with the increase in the gemcitabine concentration (P<0.001). The CCK-8 assay results showed that when the concentration of gemcitabine was 0, the proliferation ability of PANC-1-GEM cells was similar to that of PANC-1 cells; when the gemcitabine concentration was 100 nM, the proliferation ability of PANC-1-GEM cells was significantly higher than that of PANC-1 cells (P<0.001) (Figure 7A).

qRT-PCR detection of hsa_circ_0074298 expression in PANC-1-GEM-resistant cells and parental PANC-1 cells revealed that hsa_circ_0074298 expression was significantly higher in PANC-1-GEM cells than in parental cells (P<0.001) (Figure 7B), suggesting that the resistance of PANC-1-GEM cells to gemcitabine was related to the increased expression of hsa_circ_0074298.

The expression of multidrug resistance 1 (MDR1) and SMOC2 in the PANC-1, PANC1-GEM, and PANC-1-GEM+sh-circ0074298 groups was detected by Western blotting. The results showed that MDR1 and SMOC2 expression was significantly lower in the parental PANC-1 cells than in the PANC1-GEM and PANC-1-GEM+sh-circ0074298 groups (P<0.001) and that knockdown of hsa_circ_0074298 downregulated the expression of MDR1 and SMOC2 protein in PANC-1-GEM resistant cells (P<0.01) (Figure 7C), indicating that interference with hsa_circ_0074298 expression in PANC-1-GEM cells could result in a decrease in the protein expression of MDR1 and SMOC2.

PANC-1-GEM cells stably transduced with sh-circ0074298 or sh-NC were transiently transfected with miR-519d inhibitor or SMOC2 overexpression plasmids, cultured in complete medium containing 100 nM gemcitabine for 48 h, and then tested for proliferation ability using a CCK8 kit. The results showed that compared with the PANC-1-GEM cells in the NC group, the gemcitabine resistance of the PANC-1-GEM cells in the sh-circ0074298 group was significantly weakened (P<0.001); compared with PANC-1-GEM cells in the sh-circ0074298 group, the gemcitabine resistance of PANC-1-GEM cells in the sh-circ0074298+miR-519d inhibitor and the sh-circ0074298+SMOC2 overexpression groups again increased (P<0.001) (Figure 7D). These results indicated that miR-519d inhibition and SMOC2 overexpression could enhance chemoresistance.

Flow cytometry results showed that among the 4 groups, the proportion of apoptotic PANC-1-GEM cells was highest in the sh-circ0074298 group. The proportion of apoptotic PANC-1-GEM cells was lower in the sh-circ0074298+miR-519d inhibitor and sh-circ0074298+SMOC2 expression groups than in the sh-circ0074298 group (P<0.001) (Figure 7E), indicating that miR-519d inhibition and SMOC2 overexpression could reduce PANC-1-GEM cell apoptosis.

The xenograft model of PANC-1-GEM transfected with sh-NC, sh-circ007428, sh-circ007428+inhibitor (PANC-1-GEM cells stably transduced with sh-circ0074298 transiently transfected with miR-519d inhibitor), and sh-circ007428+SMOC2 (PANC-1-GEM cells stably transduced with sh-circ0074298 transiently transfected with SMOC2 overexpression) was established. Every nude mice was injected with equal volumes of gemcitabine (50 mg/kg) or PBS intraperitoneally every 2 days. Compared with intraperitoneal injection of PBS, the volume (P<0.05) and weight (P<0.01) of subcutaneous grafted tumors of intraperitoneal gemcitabine injection were decreased. The volume and weight of xenograft tumors after downregulation of hsa_circ_0074298 expression were the lowest among the 4 groups. Knockdown of hsa_circ_0074298in PANC-1-GEM cells increased PANC-1-GEM cell sensitivity to gemcitabine. The sh-circ007428+inhibitor group or sh-circ007428+SMOC2 group showed a decrease in sensitivity to gemcitabine and an increase in tumor volume and weight. The results indicated that miR-519d inhibitor or SMOC2 overexpression could effectively reverse the increased sensitivity to gemcitabine induced by downregulation of hsa_circ_0074298 expression and that drug resistance to gemcitabine was again increased (Figure 7F-H). After downregulation of hsa_circ_0074298 expression, the protein expression of MDR1 and SMOC2 in xenograft tumors significantly decreased (P<0.01). However, the miR-519d inhibitor or SMOC2 overexpression effectively reversed these effects (Figure 7I).

## Discussion

circRNAs can act as biological “sponges” of miRNA molecules to absorb miRNAs and block their inhibition of downstream target genes 4, forming a circRNA-miRNA-mRNA regulatory network. For example, the circRNA ciRS7 (CDR1as) has been shown to have more than 70 possible miRNA binding sites 7, and CDR1as functions by sponging the tumor suppressor miR-7, an interaction that has been confirmed in laryngeal squamous cell carcinoma 26, non-small cell lung cancer 27, and pancreatic cancer 28. The circRNA LDLRAD3, confirmed as a biomarker for the diagnosis of pancreatic cancer 29, regulates the progression of pancreatic cancer through the miR-137-3p/PTEN axis 30.

**Figure 8:**
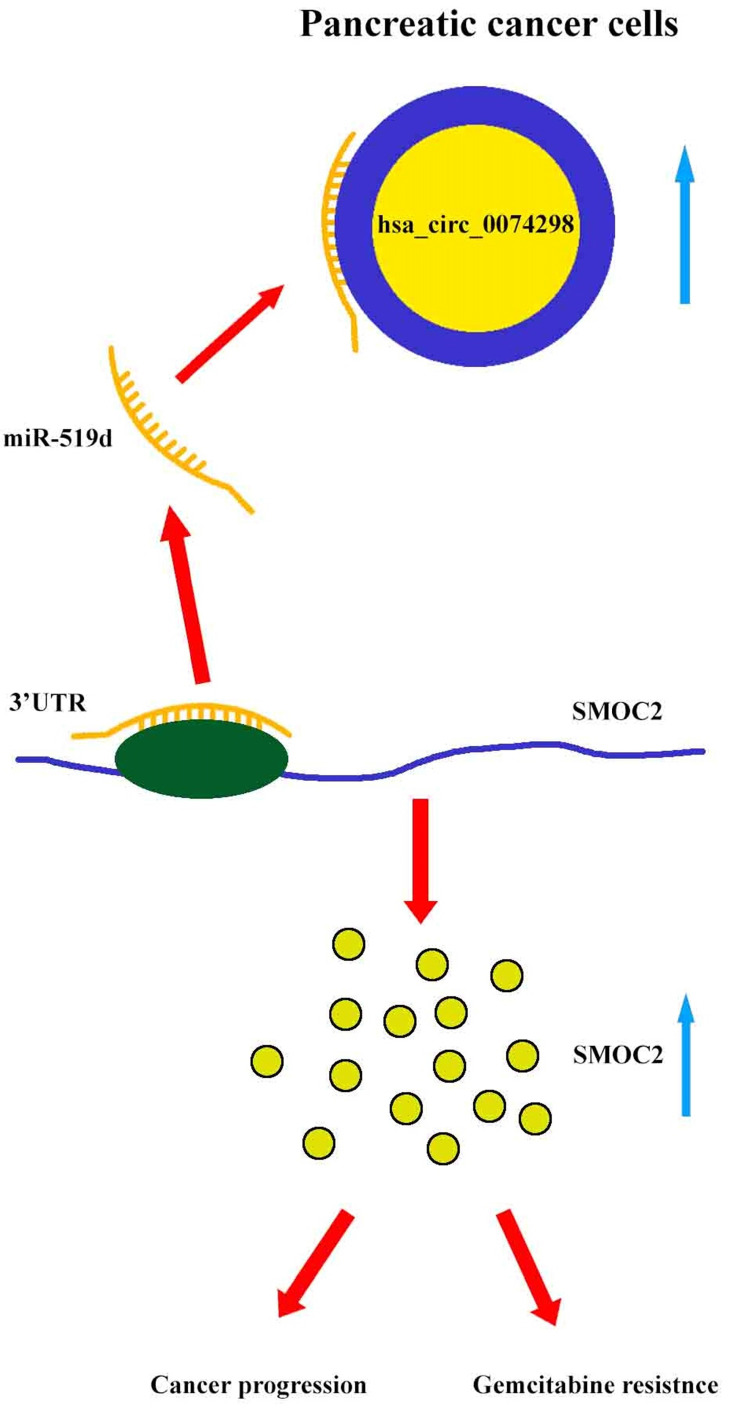
Mechanism diagram. Hsa_circ_0074298 promotes the expression of SMOC2 through the competitive binding to miR-519d in pancreatic cancer cells, thereby promoting the cancer progression and gemcitabine resistance of pancreatic tumor.

Our results showed that hsa_circ_0074298 sponged miR-519d and thus affected the downstream SMOC2 gene to promote pancreatic cancer progression and gemcitabine resistance (Figure 8).

Pancreatic cancer is one of the most common clinical tumors 31. Early diagnosis is difficult, and comprehensive treatment based on surgery does not improve the survival time and quality of life of patients after surgery 32. This study found that in human pancreatic cancer tissues, hsa_circ_0074298 comprised 1,486 bp and was produced by splicing parts of exons of the HARS gene. It is located in the cytoplasm and plays a role as a ceRNA. hsa_circ_0074298 expression in pancreatic cancer tissue was significantly higher than that in normal pancreatic tissue, and its expression level was significantly related to tumor size, tumor lymphatic metastasis, and pathological grade, indicating that the upregulation of hsa_circ_0074298 expression might be an important factor in the invasion and metastasis of pancreatic cancer.

The results of this study showed that hsa_circ_0074298 promoted the proliferation of pancreatic cancer cells, enhanced migration and invasion, increased colony formation, accelerated cell cycle progression, and reduced pancreatic cancer cell apoptosis. To further clarify the mechanism by which hsa_circ_0074298 regulates the biological behaviors of pancreatic cancer cells, a dual luciferase reporter gene assay was conducted. The results indicated that hsa_circ_0074298 and miR-519d could bind through complementary base pairing and that the expression of hsa_circ_0074298 and miR-519d was negatively correlated, suggesting that hsa_circ_0074298 might sponge miR-519d. miR-519d can posttranscriptionally inhibit SMOC2 by binding to its 3'UTR. Studies have confirmed that SMOC2 is involved in the regulation of a variety of cell biological processes, such as the cell cycle, cell proliferation, and migration 33. Studies of liver cancer 34, colon cancer 35, and thyroid cancer 36 have shown that SMOC2 can regulate malignant tumor progression, can serve as a tumor stem cell marker, and is closely related to patient prognosis. The results of *in vivo* and *in vitro* experiments in this study showed, for the first time, that SMOC2 expression was intimately correlated with the malignant progression of pancreatic cancer and that SMOC2 played a cancer-promoting effect.

Results using a stably transduced cell line with interference of hsa_circ_0074298 expression and transient transfection of miR-519d inhibitor or SMOC2 overexpression plasmids suggested that hsa_circ_0074298 played a biological role through the miR-519d/SMOC2 axis to regulate the proliferation, migration, and invasion of pancreatic cancer cells, indicating that hsa_circ_0074298 was closely associated with the malignant progression of pancreatic cancer. This mechanism is different from the mechanism by which miR-519d inhibits tumor growth through the Wnt/β-catenin pathway in pancreatic cancer 16, indicating that there are multiple miR-519d regulatory pathways in pancreatic cancer. In this study, downregulation of hsa_circ_0074298 only partially inhibited the malignant progression of pancreatic tumors, further supporting the presence of other complex regulatory mechanisms in pancreatic cancer.

Downregulating the expression of hsa_circ_0074298 increased the sensitivity of PANC-1 cells to gemcitabine. In this study, hsa_circ_0074298 was highly expressed in the gemcitabine-resistant strain PANC-1-GEM, a finding that is consistent with another report 37. After downregulating hsa_circ_0074298 expression in PANC-1-GEM cells, the protein expression of MDR1 was significantly reduced, the resistance of PANC-1-GEM cells to gemcitabine was significantly weakened, and the sensitivity to gemcitabine was improved. However, the decrease in gemcitabine resistance induced by downregulation of hsa_circ_0074298 in panc-1-GEM could be reversed by cotransfection of miR-519d inhibitor or SMOC2 overexpression, further verifying that hsa_circ_0074298 regulated pancreatic cancer through the miR-519d/SMOC2 axis *in vitro* and *in vivo*. Furthermore, downregulation of hsa_circ_0074298 promoted the apoptosis of gemcitabine-resistant cells, and retransfection of miR-519d inhibitor or SMOC2 overexpression plasmids reversed the increase in the proportion of apoptotic cells caused by the downregulation of hsa_circ_0074298.

In this study, we investigated the expression of hsa_circ_0074298 in cancer tissue and cells; however, the expression of this circRNA in human blood circulation or in the intercellular matrix was not assessed. In the future, we will study the expression of hsa_circ_0074298 in serum exosomes from pancreatic cancer patients and investigate whether serum exosomal hsa_circ_0074298 levels can be used to distinguish between chemoresistant patients and chemosensitive patients with pancreatic cancer.

In summary, we identified the hsa_circ_0074298/miR-519d/SMOC2 regulatory axis in pancreatic cancer for the first time. hsa_circ_0074298 promoted cell proliferation and migration, indicating that hsa_circ_0074298 may promote the occurrence and development of pancreatic cancer. We also showed that hsa_circ_0074298 induced gemcitabine resistance by regulating the miR-519d target gene SMOC2. These findings indicate that hsa_circ_0074298 is a potential diagnostic biomarker and can be developed into a novel screening method for pancreatic cancer and a novel target of chemoresistance.

## Supplementary Material

Supplementary figures and table.

## Figures and Tables

**Table 1:** Related circRNA primers.

Primer	Forward primer sequence(5'-3')	Reverse primer sequence(5'-3')
hsa_circ_0037207	GTTCTGCACCATCTTCAGG	TCTTGTAGTCGATCCCTGAGC
hsa_circ_0005109	GGGGCGTGTGGATTAAACTT	GAGTCTTGGGAGGGTTGTCA
hsa_circ_0003251	CAGCTTTCAGTTTCTCAAGTCAAAG	AGAAACCTGAAATCGTCCCA
hsa_circ_0048232	GACCCAGGAGCGATAACAGT	GAAGAGCTTCCTCGGGGCCT
hsa_circ_0074298	TTATTGATTATTACTGGCAAAAACG	CTATGTGGTAGCGTTTAATGTTGGT
hsa_circ_0089762	CATGTGCCATGTCGC	ACCTACGAGTACACCGACTA
GAPDH	CCAAAATCAGATGGGGCAATGCTGG	TGATGGCATGGACTGTGGTCATTCA
U6	CTCGCTTCGGCAGCACATA	AACGATTCACGAATTTGCTG

**Table 2:** qRT-PCR detection of circRNA expression in pancreatic cancer and para-cancerous tissues.

circRNA	Normal tissue (Mean ± SD)	Cancerous tissue (Mean ± SD)	FC	P
hsa_circ_0037207	1.163±0.186	4.156±3.672	3.57	<0.01
hsa_circ_0005109	1.285±0.285	3.665±3.010	2.85	<0.01
hsa_circ_0003251	1.154±0.104	3.774±2.883	3.27	<0.01
hsa_circ_0048232	1.269±0.212	3.910±3.072	3.08	<0.01
hsa_circ_0074298	1.105±0.094	5.778±6.607	5.23	<0.01
hsa_circ_0089762	1.308±0.228	3.938±2.493	3.01	<0.01

SD, standard deviation; FC, fold change.

**Table 3:** The relationship between the expression level of hsa_circ_0074298 in human pancreatic cancer tissues and the clinicopathological status of patients.

Pathological parameters	Proportion (%)	hsa_circ_0074298 expression		P
		High	Low	
Age				
≥60	17(56.7%)	7	10	0.306
<60	13(43.3%)	3	10	
Gender				
male	17(56.7%)	6	11	0.798
female	13(43.3%)	4	9	
Tumor site				
Head	18(60%)	7	11	
Body	7(23.3%)	1	6	0.732
Tail	5(16.7)	2	3	
Tumor diameter				
≤4	18(70%)	3	15	0.020
>4	12(30%)	7	5	
Pathological grade				
I	0			
I-II	3(10%)	0	3	0.566
II	14(46.7%)	5	9	
II-III	9(30%)	3	6	
III	4(13.3%)	2	2	
Lymphatic metastasis				
0	16(53.3%)	6	10	0.611
I	14(46.7%)	4	10	
II	0			

**Table 4:** Kendall correlation analysis between hsa_circ_0074298 expression in pancreatic cancer tissue and clinicopathological data of the patients.

Parameters	hsa_circ_0074298 expression	P
	Kendall correlation coefficient (τ)	
Age	-0.074	0.636
Gender	0.033	0.861
Tumor diameter	0.408	0.024
Lymphatic metastasis	-0.12	0.518
Pathological grade	0.433	0.012

**Table 5:** Multivariate analysis of hsa_circ_0074298 expression in pancreatic cancer tissue and clinicopathological data of the patients.

Parameters	hsa_circ_0074298 expression	P
	Z value	
intercept	-1.287	0.198
Age	1.364	0.172
Gender	0.151	0.88
Tumor diameter	4.887	<0.001
Lymphatic metastasis	-5.438	<0.001
Pathological grade	3.558	<0.001
