# Supplementary material for: Hsa_circ_0074298 promotes pancreatic cancer progression and resistance to gemcitabine by sponging miR-519 to target SMOC
Source: J Cancer. 2022 Jan 1;13(1):34–50. doi: 10.7150/jca.62927 (PMC8692684; doi:10.7150/jca.62927)
Supplement: Supplementary file 1 — Supplementary figures and table. [file jcav13p0034s1.pdf]

Method

RNase R Treatment Assay

2 mg total RNA was incubated with or without 3 U/mg of RNase R (Epicenter Technologies) for 30 min at 37°C. Then, qRT-PCR was performed to detect the expression levels of has_circ_0074298 and linear β-actin as negative control.

Table S1 miRNA binding sites to hsa_circ_0074298 (pairing sites marked as red)

miRNA	Binding site
hsa-miR-519d	5'- CAAAGUGCCUCCCUUUAGAGUG-3'
hsa-miR-582-3p	5'- UAACUGGUUGAACAACUGAACC-3'
hsa-miR-510	5'- UACUCAGGAGAGUGGCAAUCAC-3'
hsa-miR-497	5'- CAGCAGCACACUGUGGUUUGU-3'
hsa-miR-503	5'- UAGCAGCGGGAACAGUUCUGCAG-3'
hsa-miR-515-3p	5'- GAGUGCCUUCUUUUGGAGCGUU-3'
hsa-miR-532-3p	5'-CCUCCCACACCCAAGGCUUGCA-3'
hsa-miR-572	5'-GUCCGCUCGGCGGUGGCCCA-3'
hsa-miR-578	5'- CUUCUUGUGCUCUAGGAUUGU -3'
hsa-miR-548p	5'- UAGCAAAAACUGCAGUUACUUU -3'
hsa-miR-615-5p	5'- GGGGGUCCCCGGUGCUCGGAUC -3'
hsa-miR-769-3p	5'- CUGGGAUCUCCGGGGUCUUGGUU -3'

Figure S1 Detection of hsa_circ_0074298 expressions before and after RNase R treatment by qRT-PCR

PANC-1

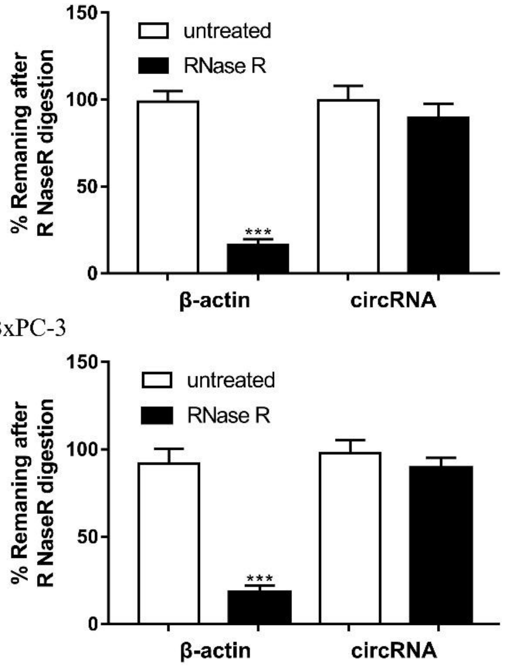

Treatment of RNase R to confirm the circular characteristics of has_circ_0074298. (***P<0.001)

Figure S2 Western blot of HARS expression affected by hsa_circ_0074298 knockdown

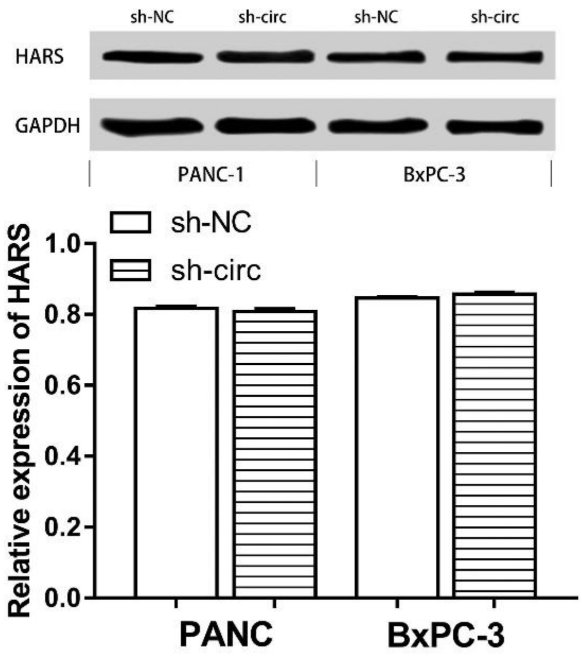

The impact of sh-circ0074298 on the expression of HARS protein in PANC-1 and BxPC-3 cells.
